# Ultrasonic-Assisted Extraction of Xanthorrhizol from *Curcuma xanthorrhiza* Roxb. Rhizomes by Natural Deep Eutectic Solvents: Optimization, Antioxidant Activity, and Toxicity Profiles

**DOI:** 10.3390/molecules29092093

**Published:** 2024-05-01

**Authors:** Adelina Simamora, Kris Herawan Timotius, Heri Setiawan, Febrina Amelia Saputri, Chinthia Rahadi Putri, Dewi Aryani, Ratih Asmana Ningrum, Abdul Mun’im

**Affiliations:** 1 Graduate Program of Pharmaceutical Sciences, Faculty of Pharmacy, Universitas Indonesia, Depok 16424, West Java, Indonesia; adelina.simamora@ukrida.ac.id; 2 Department of Biochemistry, Faculty of Medicine and Health Sciences, Krida Wacana Christian University, Jakarta 11510, Indonesia; kh_timotius@ukrida.ac.id; 3 National Metabolomics Collaborative Research Center, Faculty of Pharmacy, Universitas Indonesia, Depok 16424, West Java, Indonesia; heri.setiawan@farmasi.ui.ac.id (H.S.); ratih.asmana@gmail.com (R.A.N.); 4 Department of Pharmacology, Faculty of Pharmacy, Universitas Indonesia, Depok 16424, West Java, Indonesia; 5 Faculty of Pharmacy, Universitas Indonesia, Depok 16424, West Java, Indonesia; febrina.amelia@farmasi.ui.ac.id (F.A.S.); chinthia.rahadi@ui.ac.id (C.R.P.); dewi.aryani81@ui.ac.id (D.A.); 6 Research Center for Genetic Engineering, Research Organization for Life Sciences and Environment, National Research and Innovation Agency (BRIN), Cibinong, Bogor 16911, West Java, Indonesia; 7 Department of Pharmacognosy-Phytochemistry, Faculty of Pharmacy, Universitas Indonesia, Depok 16424, West Java, Indonesia

**Keywords:** DNA protection activity, NADES, NOESY, response surface methodology, scanning electron microscopy, stability, xanthorrhizol

## Abstract

Xanthorrhizol, an important marker of *Curcuma xanthorrhiza*, has been recognized for its different pharmacological activities. A green strategy for selective xanthorrhizol extraction is required. Herein, natural deep eutectic solvents (NADESs) based on glucose and organic acids (lactic acid, malic acid, and citric acid) were screened for the extraction of xanthorrhizol from *Curcuma xanthorrhiza*. Ultrasound-assisted extraction using glucose/lactic acid (1:3) (GluLA) gave the best yield of xanthorrhizol. The response surface methodology with a Box–Behnken Design was used to optimize the interacting variables of water content, solid-to-liquid (S/L) ratio, and extraction to optimize the extraction. The optimum conditions of 30% water content in GluLA, 1/15 g/mL (S/L), and a 20 min extraction time yielded selective xanthorrhizol extraction (17.62 mg/g) over curcuminoids (6.64 mg/g). This study indicates the protective effect of GluLA and GluLA extracts against oxidation-induced DNA damage, which was comparable with those obtained for ethanol extract. In addition, the stability of the xanthorrhizol extract over 90 days was revealed when stored at −20 and 4 °C. The FTIR and NMR spectra confirmed the hydrogen bond formation in GluLA. Our study reported, for the first time, the feasibility of using glucose/lactic acid (1:3, 30% water *v*/*v*) for the sustainable extraction of xanthorrhizol.

## 1. Introduction

**Figure 1:**
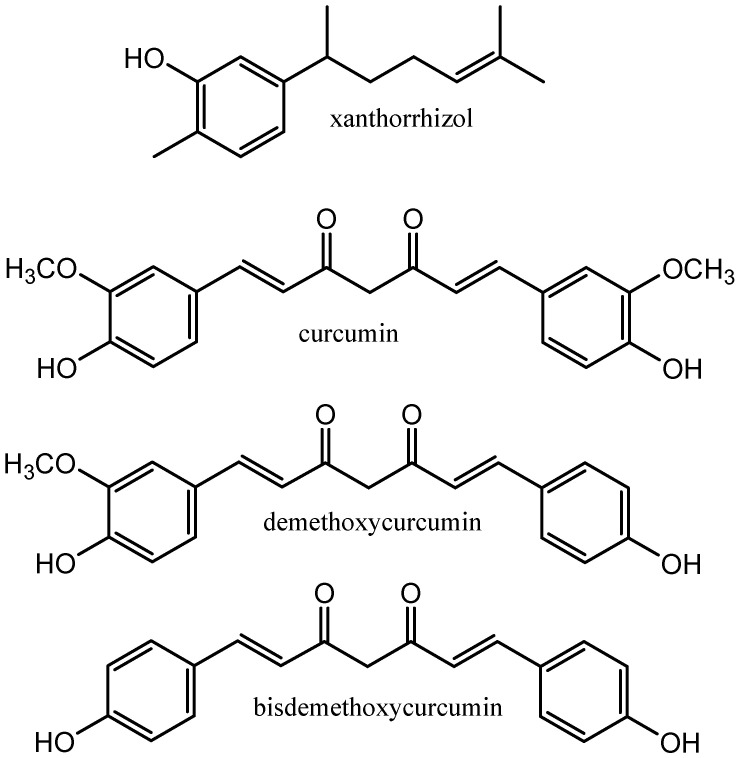
Targeted compounds extracted from rhizomes of *Curcuma xanthorrhiza*.

*Curcuma xanthorrhiza* of the family Zingeberaceae is a rhizomatous plant that originates from Indonesia and is cultivated throughout tropical areas. The rhizomes of *C. xanthorrhiza* have been popularly used in Indonesian traditional medicine for a long time as a tonic and for the treatment of different diseases, including liver and stomach diseases [1]. Various bioactive compounds have already been identified in *C. xanthorrhiza* rhizomes, namely diarylheptanoids, phenolics, and terpenoids [2]. Of these, xanthorrhizol, which is a bisabolene sesquiterpenoid, is found to be the major bioactive compound in the rhizome [3]. Xanthorrhizol has received attention for its wide-ranging biological activities such as anticancer [4], antimicrobial [5], anti-hyperglycemia [6], anti-inflammatory, and antioxidant activities [7]. Therefore, it is important to develop an efficient extraction method for xanthorrhizol. In addition to xanthorrhizol, curcuminoids (Figure 1) are present in significant abundance in the rhizome of *C. xanthorrhiza* [8]. Various studies have reported the use of natural deep eutectic solvents (NADESs) for the extraction of curcuminoids from different curcuma species [9,10]. However, the selective extraction of xanthorrhizol over curcuminoids has not been reported yet.

To date, the extraction of xanthorrhizol has mostly been performed based on conventional techniques, such as maceration, percolation, and Soxhlation [6,11]. Organic solvents are largely applied for this purpose. The use of organic solvents causes several issues as they are often toxic, flammable, explosive, and nonbiodegradable and, thus, hazardous to health and the environment.

Growing concern for the environment and the need to obtain less contaminated products due to hazardous residues of organic solvent have stimulated the development of greener extraction processes. Recently, natural deep eutectic solvents have gained recognition as an alternative to organic solvents. First reported by Abbot et al. [12], deep eutectic solvents consisted of hydrogen bond acceptor (HBA) and hydrogen bond donor (HBD) components, which, upon mixing, bring about the formation of hydrogen bond interactions among components, leading to a liquid eutectic mixture. Different combinations of HBA and HBD components allow for the adjustment of solvent affinity to extract specific bioactive compounds. NADESs have been successfully applied for the extraction of different types of bioactive compounds, including flavonoids [13], anthocyanins [14], phenolics [15], and triterpenes [16].

**Figure 2:**
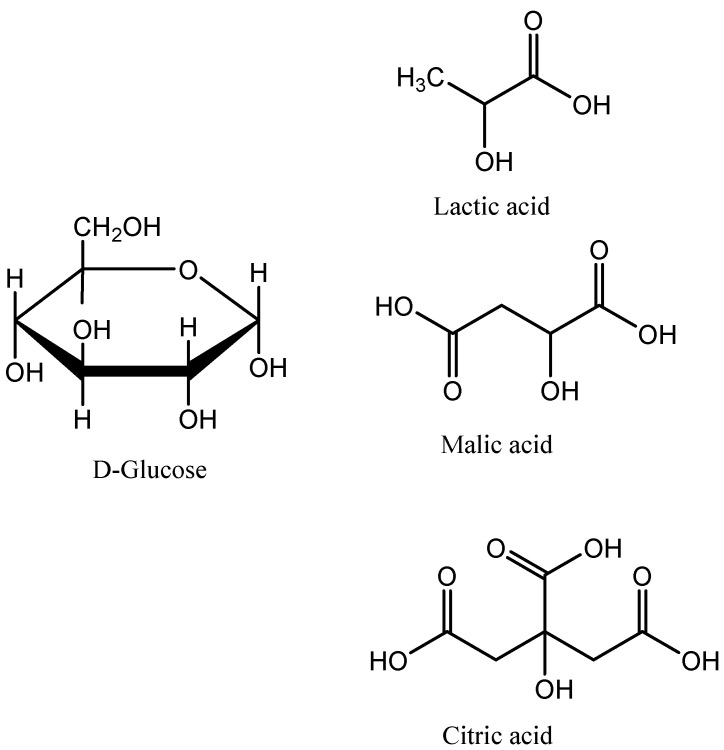
NADES components used in the present study.

Due to the high pharmacological potency of xanthorrhizol, together with its high abundance in the rhizomes of *C. xanthorrhiza* (accounting for 64.38% in the rhizome oil) [11] and potential nutraceutical application, we proposed the use of NADESs as an alternative solvent for the selective extraction of xanthorrhizol from the rhizomes of *C. xanthorrhiza*. In the present study, three types of NADESs using glucose as a hydrogen bond acceptor and organic acids (lactic acid, malic acid, and citric acid) as hydrogen bond donors (Figure 2) were synthesized, characterized, and used in the extraction. Following the identification and characterization of the most promising NADESs, optimization of the extraction was conducted using the response surface methodology (RSM) by optimizing the extraction parameters, namely the solid-to-liquid (S/L) ratio, water content, and duration of extraction. NMR and SEM analyses were conducted to reveal the mechanisms of extraction by NADESs. A comparative study concerning the extraction yields, phytochemical profiles, protection effect against DNA damage, and stability of xanthorrhizol extracts was conducted via a conventional extraction method using ethanol (96%).

## 2. Results and Discussion

### 2.1. NADES Preparation

In the present study, three glucose-based NADESs were evaluated for the extraction of xanthorrhizol from rhizomes of *C. xanthorrhiza*. In these NADES systems, glucose was combined with lactic acid, malic acid, and citric acid, each with a molar ratio of 1:3. The physicochemical properties of the studied NADESs are shown in Table 1. To allow for a comparison, all NADESs were prepared with the addition of the same amount of water (20%).

**Figure 3:**
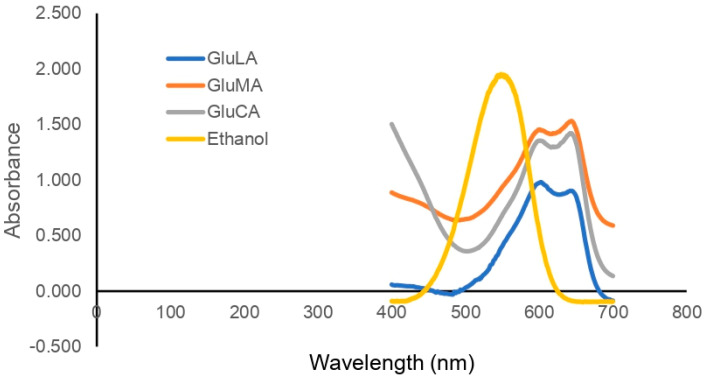
The UV–Vis spectra of the Nile red solvatochromic probe in different NADESs.

The polarity of DESs can be determined indirectly by measuring the *K*_max_ of the Nile red indicator in different solvents [17]. More polar solvent shifts the λ_max_ of the Nile red to a longer wavelength, thus lowering E_NR_. The results in Table 1 and Figure 3 show a slight variation in the polarity of the glucose-based NADESs, in the following order: GluLA ≈ GluMA > GluCA. These results suggest that the acidity of organic acids did not significantly influence the polarity of the studied NADESs.

Under the same measurement conditions, the studied NADESs were highly viscous (Table 1). The viscosity of DESs originates from the hydrogen bond interaction between components. The organic acids used in this study allow for maximum interaction with glucose due to the HBAs, with LA, MA, and CA containing mono-, di-, and tri-carboxiclic acid groups, respectively. GluMA and GluCA achieved viscosity of >49.152 mPa·s. Glucose based NADESs are known to be very viscous, as reported by Mitar et al. (2019) [18]. A viscous solvent may restrict mass transfer in the extraction process, which leads to lower extraction efficiency. However, high solvent viscosity allows for a stable molecular interaction [18].

Density is an important property of solvents. Generally, solvents with high density are more difficult to handle and mix in the chemical process. However, the selection of highly dense solvents can be beneficial to ensure phase separation in the extraction process. In the present study, the density of the glucose-based NADESs with 20% water (*v*/*v*) was determined, and the values range from 1.31–1.46 (Table 1), which are higher than those of water and ethanol, with the following order: GluCA > GluMA > GluLA. Mitar et al. (2019) found that density is a property that shows an additive relationship among their components [18]. With the same HBA (glucose), the order of density is likely due to the lengthening of the alkyl chain and the addition of the carboxylic group in the HBA of NADESs. In another study, it was also observed that density increases with the number of -OH groups present in the compounds [19], which is consistent with what was observed in this study.

The combination of glucose and lactic acid (1:1) was previously reported to be able to extract curcuminoids from *C. longa* with good yield [10]. In the present study, a different molar ratio and different dicarboxylic acids were used to allow modification of the polarity of synthesized NADESs to accommodate xanthorrhizol extraction over curcuminoids [20,21]. Each NADES was prepared through heating and stirring to obtain clear eutectic mixtures. It should be noted that in the process of NADES syntheses, no chemical reaction took place between the starting components. This indicates that their synthesis is highly efficient, produces no waste, and has a low cost.

### 2.2. Evaluation of NADES Antioxidative Activity

The antioxidative activity of NADESs is less known, and limited reports are available regarding this activity [22]. It is well known that the components forming NADESs such as lactic acid, malic acid, and citric acid, have antioxidant activity. Therefore, in this study, the antioxidant activity of glucose-based NADESs with these organic acids was evaluated. DPPH and FRAP assays were employed to evaluate the antioxidative capacity of the studied NADESs.

The DPPH radical-scavenging activity of the NADESs studied herein was between 50.42 and 60.69 µG AAE g^−1^ (Table 1). The activity was much higher than the organic solvent ethanol (6.53 µG AAE g^−1^). The antioxidant activity of NADESs is expected to be due to the components forming NADES (lactic acid, malic acid, and citric acid), which are known to have radical-scavenging activity.

The FRAP assay is widely used for quantifying the antioxidant capacity of plant extracts. Ferrous ion (III) is reduced to a lower oxidation number in acidic conditions by antioxidant compounds in samples. Among the tested NADESs, GluCA exhibited the best ability to reduce Fe(III), followed by GluLA, whereas the lowest FRAP value was observed for GluMA (Table 1). This is possibly due to citric acid, which is well known in the literature to be a strong antioxidant.

### 2.3. Evaluation of NADES Toxicity

NADESs are considered safe, eco-friendly, and benign since their starting components are of natural origin and are found in living organisms. This implies cellular tolerance and low toxicity in living organisms. However, some reports showed the opposite effects of NADESs [23], which indicates that the toxicity profile of the final mixture of NADESs can be different from their individual components, possibly due to the synergistic effect between components [24]. In addition, although NADESs have the advantage of being sustainable solvents, their toxicity in the environment remains to be confirmed. Studies regarding the toxicity of NADESs have been conducted to a lesser extent, using different organisms such as vertebrates, invertebrates, and cell line models [25]. In the present study, the toxicity of the studied NADESs was assessed using bacteria as model organisms. A bacterial time-kill assay was employed in this study, which is advantageous over other bacterial assays such as disk test assays as the time-kill assay is simple and allows real-time analysis.

To analyze the tolerance of *E. coli* and *S. aureus* to the studied NADESs and, thus, the potential toxicity of NADESs, cells were grown in LB media containing different NADESs (GluLA, GluMA, and GluCA), and the growth was monitored continuously overnight in real time. The results were compared with those of the control.

**Figure 4:**
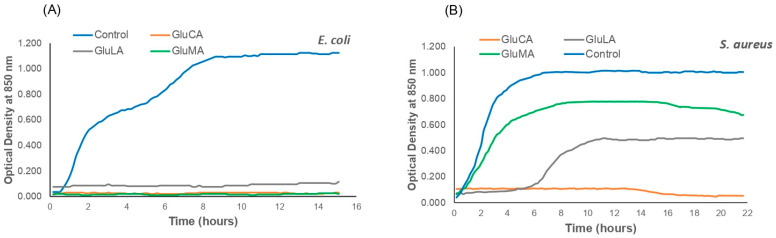
The impact of the studied NADESs (GluLA, GluMA, and GluCA) on the growth of (**A**) *E. coli* and (**B**) *S. aureus*. Each NADES contained 20% (*v*/*v*) water.

The *E. coli* growth curves in Figure 4A show two different patterns. The control culture (*E. coli* without NADES) showed a bacterial growth pattern. A short lag phase (adaption period) was observed within 5 min of incubation, followed by an exponential growth phase (log phase) which took place for 8 h; thereafter, this was followed by a stationary phase. It is notable that in the present study, *E. coli* shows diauxic curves in which two growth curves were observed to have a short lag phase. Diauxic growth curves have been reported for *E. coli* and many other bacteria previously [26]. In contrast, different patterns were shown by *E. coli* treated with GluLA, GluMA, and GluCA, in which cell growth was not detected, indicating the toxicity of GluLA, GluMA, and GluCA for *E. coli*.

Figure 4B shows the growth curves of *S. aureus* in LB media containing GluLA, GluMA, and GluCA. The control culture shows the expected growth pattern with a short lag phase followed by a fast growth rate that yields high biomass, with the final OD value reaching around 1, and thereafter followed by a stationary phase. However, unlike *E. coli*, the treatment of GluLA and GluMA in *S. aureus* did not inhibit bacterial growth. In the case of GluMA, the curve shows similar growth to the control. The lag and log phases occur at a similar incubation time; however, a decreased growth rate is observed for GluMA, yielding a lower final OD of around 0.6 (40% lower than the control). A different growth profile is observed for GluLA. *S. aureus* experienced a prolonged lag phase of about a 6 h incubation period. Following the treatment with GluLA, the bacterium grew at a much slower rate, as can be seen in the log phase, reaching a final OD of 0.4 (60% lower than control). In the case of GluCA, treatment in *S. aureus* caused cell death, as also observed in *E. coli*.

The outer membrane of Gram-negative bacteria, which is the lipopolysaccharide (LPS) layer, is generally negatively charged [25,27]. The stabilization of the LPS is due to the presence of divalent cations within the LPS core, which form electrostatic cross-links with phosphate groups in the LPS core. This reduces the electrostatic repulsion between membrane groups. In the present study, 50 µL of each NADES, i.e., GluLA and GluMA (each with pH 5–6 in LB media) and GluCA (pH 3–4 in LB media), was added to 15 mL of *E. coli* culture in LB media. The amount of water that dominates the surroundings of these NADESs disrupts the hydrogen bond network between components in NADESs, releasing the anionic lactate, malate, and citrate. These anions may chelate the divalent metal ions, leading to the destabilization of the LPS layer. The antibacterial activity of citric acid is well known, and a previous study showed membrane damage using a scanning electron micrograph (SEM) due to low pH [27]. LPS disruption may also be due to the acidification of the intracellular compartment. Small uncharged organic acids which are more lipophilic may also permeate the LPS membrane and releasing protons to the intracellular environment, collapsing the proton gradient [28].

### 2.4. NADES Extraction of Xanthorrhizol and Determination of the Optimal Conditions Using an Ultrasound-Assisted Extraction Process

The response surface methodology (RSM) with a Box–Behnken Design (BBD) was applied in the optimization of xanthorrhizol extraction. The independent variables for RSM, which were selected based on our preliminary studies, were water content (10–30%), solid-to-liquid ratio (1/5 to 1/20 g/mL), and extraction time (10–30 min) (Supplementary Table S1). The experimental responses used in the present study were xanthorrhizol and curcuminoid contents, which were analyzed using a validated method of thin layer chromatography (TLC) with densitometry. The BBD and experimental values of xanthorrhizol and curcuminoid contents are listed in Table 2. The final xanthorrhizol content as a function of three independent variables (water content, solid-to-liquid ratio, and extraction time) is described by the following polynomial equation:

Y = 11.8666 + 0.98X_1_ + 2.86X_2_ − 1.03X_3_ + 5.13X_1_X_2_ + 0.51X_1_X_3_ − 1.07 − 0.75X_1_^2^ − 2.69X_2_^2^ − 6.34X_3_^2^ (1)

For the determination of the response variable of curcuminoid content, the following equation was obtained:

Y = 4.64101 − 0.12X_1_ + 1.70B + 0.11C + 0.45X_1_X_2_ − 0.08X_1_X_3_ − 0.38X_2_X_3_ − 0.04X_1_^2^ + 0.001X_2_^2^ − 0.08X_3_^2^ (2)

where Y is xanthorrhizol or curcuminoid content, and X_1_, X_2_, and X_3_ represent the water content in GluLA, the solid-to-liquid ratio, and the extraction time, respectively. The suitability of the quadratic polynomial equations was analyzed as shown in Supplementary Table S2. 

It is noteworthy that for the xanthorrhizol model, the *p* value of the “Model” is <0.0001, the *p* value of the “Lack of Fit” is not significant, and the *R*^2^ is 0.9975; meanwhile, all the linear coefficients (X_1_, X_2_, and X_3_), quadratic coefficients X_3_^2^, and partial cross coefficients (X_1_X_2_, X_1_X_3_, and X_2_X_3_) are significant (*p* < 0.05), and only the quadratic coefficients X_1_^2^ and X_2_^2^ are not significant (*p* > 0.05). The suitability of the quadratic equations for curcuminoids can be seen in Supplementary Table S2.

Based on the regression model, the predicted optimum conditions for the extraction of xanthorrhizol by ultrasound-assisted extraction with GluLA are 30% water addition to GluLA, 1/15 (g/mL), and a 20 min extraction time. Extraction using these optimal conditions obtained 17.62 ± 0.20 mg/g dried rhizome for xanthorrhizol, and 6.64 ± 0.05 mg/g dried rhizome for curcuminoids. The experimental values for the xanthorrhizol and curcuminoid contents are 21.75 ± 0.06 and 6.79 ± 0.10 mg/g, which are close to the predicted value, indicating good accuracy of the final xanthorrhizol and curcuminoid contents. These results signify the selectivity of the optimized extraction for xanthorrhizol over curcuminoids. To the best of our knowledge, this is the first time NADESs have been reported for the extraction of xanthorrhizol.

### 2.5. Xanthorrhizol Extraction by GluLA and Comparison with Ethanol

#### 2.5.1. Ethanol Maceration

As previously mentioned, ethanol maceration is the method recommended by the Indonesian Farmakope for the extraction of xanthorrhizol from rhizomes of *C. xanthorrhiza*. In the case of ethanol maceration, the yields obtained were 9.14 ± 0.01 mg/g and 2.37 ± 0.01 mg/g for xanthorrhizol and curcuminoids, respectively. The comparison of extraction yields between ultrasound-assisted NADESs with the ethanol maceration method highlights the efficiency of extraction by UAE-NADESs in terms of extraction time (20 min in comparison with several days). Although GluLA was more viscous than ethanol and, thus, may limit mass transfer in the extraction process, the use of ultrasonication aided in the extraction process. The extraction efficiency of using combined UAE-NADESs was reported previously by Patil et al. (2021) [20].

#### 2.5.2. Surface Morphology Analysis

The structural changes in the surface morphology of *C. xanthorrhiza* rhizome powder were investigated using scanning electron microscopy (SEM) to reveal the effect of GluLA and ultrasonication on the raw material. Comparisons were made between untreated samples and samples soaked in ethanol.

**Figure 5:**
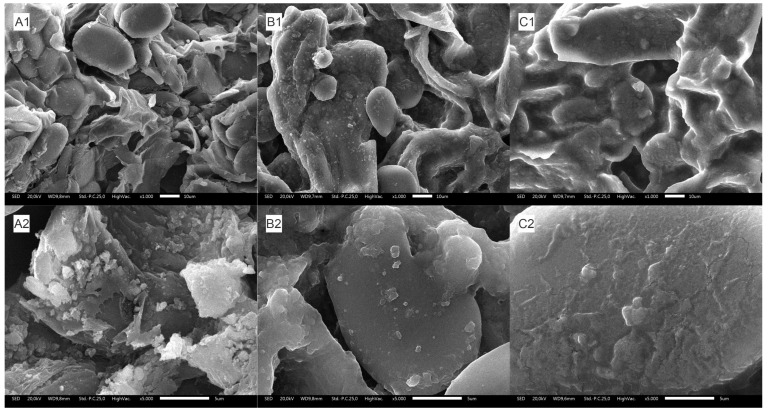
SEM images of rhizome powder of *C. xanthorrhiza* taken at magnifications of 1000 and 5000. (**A1**,**A2**) Untreated powder; (**B1**,**B2**) powder after extraction using EtOH; (**C1**,**C2**) powder after extraction using Glu:LA (1:3).

Untreated rhizome powder showed an intact and smooth surface (Figure 5(A1,A2)). The surface of the rhizome particle did not change appreciably following extraction with ethanol (Figure 5(B1,B2)). By comparison, the external rhizome surface treated with GluLA (Figure 5(C1,C2)) exhibited a different morphology. The rhizome particle showed a loose, disintegrated, and cracked surface. This structural damage can be attributed to sonoporation due to the combined use of GluLA and exposure to ultrasonication in the extraction.

Ultrasonic wave vibration in GluLA media propagates the formation of cavitation bubbles. Although viscous solvents such as NADESs may need higher energy to induce the formation of cavitation bubbles, their low-vapor-pressure characteristics produce more intense bubble collapse [29], giving rise to stronger local turbulence. The mechanical effect of bubble implosion may aid in the disruption and disintegration of the rhizome surface to allow the mass transfer of phytocompounds to the bulk solvent.

#### 2.5.3. Metabolite Identification of GluLA and Ethanol Extracts

**Figure 6:**
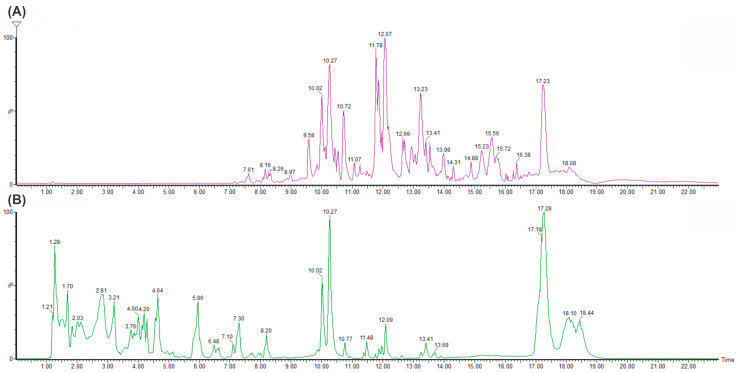
LC-MS/QTOF chromatograms of extracts of *C. xanthorrhiza* at *m*/*z* 50–1000. (**A**) Ethanol extract; (**B**) GluLA extract.

An analysis of the chemical constituents in GluLA and ethanol extracts of *C. xanthorrhiza* was carried out in LC-MS/QTOF experiments. The chromatograms of the extracts are shown in Figure 6. Table 3 presents information on the peaks observed and putative identification of the compounds, with a mass error of ±10 ppm, which indicates good mass accuracy of the compounds identified in the mass spectra. These compounds were tentatively identified by interpreting the chemical formula of the molecular ions [M − H]^+^, fragmentation patterns, and the elution order. This information was compared with available data in the literature documenting previous identification of the compounds. Public mass databases such as Mass bank, NIST, PubChem, and ChemSpider were also used for comparison. Moreover, literature regarding the phytochemical profiles of the rhizomes of *C. xanthorrhiza* were used as indicative information for component identification [2,30,31,32].

The LC-MS/QTOF analysis of GluLA and the ethanol extracts confirms the presence of well-known compounds previously reported in the rhizome of *C. xanthorrhiza*. The identified compounds herein belong to different groups, such as terpenoids, diarylheptanoids, flavonoids, and phytosterols (Table 3). Generally, different chromatogram profiles were obtained for GluLA and ethanol extracts (Figure 6). The GluLA profile (Figure 6B) shows more peaks in the retention time (RT) (4.04 to 7.30) than ethanol extract (Figure 6A). Most of them are attributed to the different terpenoids. On the other hand, ethanol extract exhibits peaks in the range of RT 14 to 17. Most compounds in this range correspond to various diarylheptanoids. This discrepancy indicates the difference in polarity of GluLA and ethanol, resulting in different constituents extracted in the corresponding solvents.

#### 2.5.4. DNA Damage Protection Activity of GluLA and Ethanol Extracts

The double-strand cleavage of plasmid DNA by highly reactive •OH radicals generated by the Fenton reaction can mimic what occurs in a biological system. In the presence of H_2_O_2_/FeSO_4_, •OH radicals were formed due to electron transfer involving the oxidation of Fe(II) to Fe(III). These radicals induce the cleavage of supercoiled DNA (sc-DNA), resulting in an open circular (oc-DNA) and linear (lin-DNA) conformation with lower electrophoretic movement [33]. The separation of these conformations can be observed in the gel electrophoreses. The DNA protection assay is well known as a biomarker to evaluate the antioxidant activity of plant extracts.

**Figure 7:**
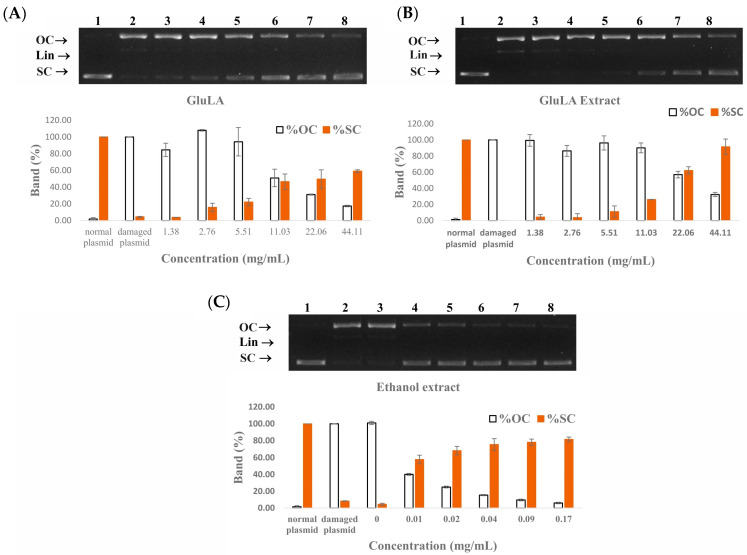
The protective effects of GluLA (**A**), GluLA extract (**B**), and ethanol extract (**C**) against H_2_O_2_-induced plasmid pBR322 DNA damage. Lanes 1 and 2 are the normal and damaged pBR322 plasmid, respectively. Lanes 3 to 8 are plasmids treated with samples of different concentrations: GluLA and GluLA extract at 1.38–44.18 mg/mL, and ethanol at 0.01–0.17 mg/mL. OC: open circular form, Lin: linear form, SC: supercoiled form.

A DNA protection assay was conducted at different concentrations of GluLA, GluLA extract, and ethanol extract. The results are presented in Figure 7. Lane 1 shows a normal plasmid in which the supercoiled form was predominant in the absence of H_2_O_2_ and Fe(II) ions. This conformation is characterized by high electrophoretic mobility. Lane 2 shows damaged DNA due to OH• radicals from the Fenton reaction. DNA was converted into the open circular (op-DNA) and linear (lin-DNA) forms which moved slower in the electrophoretic gel. Lanes 3 to 8 show DNA treated with samples in increasing concentrations. The addition of GluLA in the range of 1.38–44.18 mg/mL (lanes 3 to 8) suppressed the formation of lin-DNA and oc-DNA, and induced the partial recovery of sc-DNA, in a dose-dependent manner. The DNA-protective effect of GluLA is likely caused by the radical-scavenging activity and reducing capacity of GluLA, as shown in Table 1. In addition, the ability of LA to chelate Fe(II), thus inhibiting the Fenton reaction, may also contribute to the DNA-protective effect of GluLA. Similar to GluLA, GluLA extract showed a protection effect against DNA damage in the same concentration range as GluLA. This result indicates a contributory factor of GluLA to this activity. It should be noted, however, that ethanol extracts exerted DNA protection activity in lower concentrations (0.01–0.17 mg/mL) than GluLA and GluLA extract. Phenolics extracted from different plants have been reported for their ability to prevent breakages of plasmid DNA strands [34].

The band intensity was further analyzed by ImageJ V1.8.0. software to obtain a quantification of oc-DNA compared to sc-DNA. The results in Figure 7 confirm that GluLA is effective in protecting DNA and inhibiting strand breakage due to OH radicals. The band analysis shows that at 22.06 and 44.11 mg/mL, GluLA increased the native form of DNA by 49.51 and 59.01%, respectively. On the other hand, GluLA extract at the same concentrations retained the native DNA by 61.18 and 87.83%. Ethanol extract was able to protect DNA effectively, and at 0.02 mg/mL, retained more than 70% of the native DNA. These results highlight the antioxidative activity of the extracted bioactive compounds, including xanthorrhizol and curcuminoids.

The use of antioxidants to protect DNA strands from breakage is beneficial to suppress oxidative damage, thus potentially preventing some diseases, including cancer and degenerative diseases. The findings obtained in the present study suggest a potential health benefit of *C. xanthorrhiza* extracts in preventing health risks posed by oxidative damage to DNA.

#### 2.5.5. Stability of Xanthorrhizol Extracted by GluLA and Ethanol

Xanthorrhizol is susceptible to storage conditions, such as light exposure and length of storage. As per the information from the product data sheet, it was advised that xanthorrhizol standard compound be stored at −20 °C away from light. To date, no study has reported the stability of xanthorrhizol in extracts. To allow for further applications of xanthorrhizol-rich NADES extract, it is important to study the stability of the extracted xanthorrhizol in different storage conditions. The present study investigated the stability of xanthorrhizol in GluLA at −20, 4, and 25 °C over a 90-day storage period. The results are shown in Figure 6.

**Figure 8:**
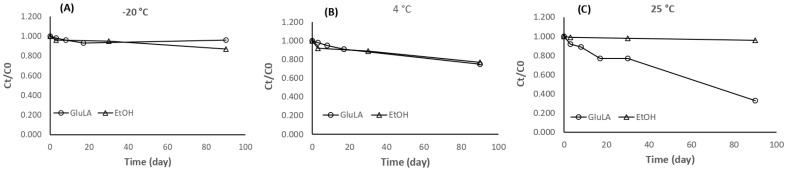
Stability of the extracted xanthorrhizol in glucose/lactic acid (1:3) and ethanol over 90 days of storage at (**A**) −20, (**B**) 4, and (**C**) 25 °C. Ct is the concentration of xanthorrhizol at *t* hours, whereas C0 is the concentration of xanthorrhizol at the start of the experiment.

Xanthorrhizol showed similar stability in GluLA and ethanol extracts at storage temperatures of −20 °C and 4 °C (Figure 8A,B). At −20 °C, the extracted xanthorrhizol in GluLA remained stable over a period of 90 days with only 4% degradation observed. Higher degradation of xanthorrhizol of 13% was observed, however, for ethanol extract at −20 °C over the same period. A storage temperature of 4 °C resulted in higher degradation compared to −20 °C, observed for both GluLA and ethanol extracts, each by 15 and 13%, respectively. Different results were obtained when the extracts were stored at 25 °C (Figure 8C). The extracted xanthorrhizol in GluLA extract was not stable at this temperature, with only 33% remaining xanthorrhizol in the GluLA extract at the end of the assay. Apparent degradation occurred after day 30. Interestingly, xanthorrhizol was stable in ethanol extract, with 96% of xanthorrhizol still retained after 90 days.

The above results indicate that storage temperature plays a significant role in the stability of xanthorrhizol in the extract. It is likely that low temperature restricts the movement of xanthorrhizol molecules to inhibit their exposure to oxidative species. With regard to the extraction solvent, xanthorrhizol in GluLA showed higher stability than ethanol extract when stored at −20 °C, indicating the contributing effect of GluLA to the observed stability.

This stabilization may have originated from the hydrogen bonding interaction between xanthorrhizol and GluLA, as is described in the previous section. The establishment of hydrogen bond formation could restrict the movement of xanthorrhizol molecules, thus limiting their contact with oxidative species. The π-π stacking between phenolic rings of xanthorrhizol may also hold the molecules stable.

Previously, Dai et al. (2013 and 2014) studied the mechanisms of stabilization of quercetin in choline chloride-xylitol. Using 2D-NMR and FT-IR spectra, it was found that the extensive hydrogen bonding interaction between solutes and NADESs greatly increase their storage stability [35,36].

#### 2.5.6. FTIR and NMR Characterization of the Optimal NADESs and Interaction of NADESs with Xanthorrhizol

**Figure 9:**
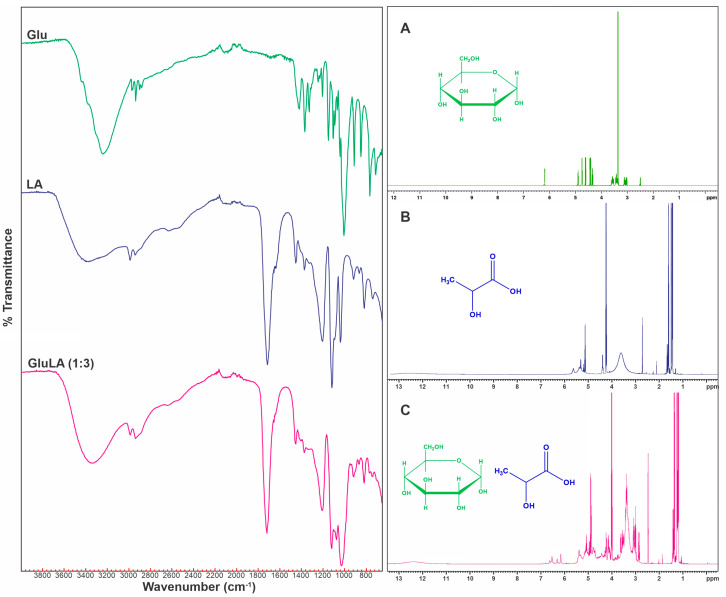
FTIR and NMR spectra of (**A**) glucose, (**B**) lactic acid, and (**C**) GluLA (1:3, 30% *v*/*v*).

FT-IR spectroscopy is a typical technique to identify H-bond interaction in a system [37]. In NADES syntheses, the formation of H-bonding between HBD and HBA components of NADESs is the main force of intermolecular interactions between components. Therefore, FT-IR spectroscopy was applied to evaluate the evidence of NADES formation. The FT-IR spectra of GluLA and its components, i.e., glucose and lactic acid, are shown in Figure 9A. The GluLA spectrum was dominated by lactic acid functional groups. The O-H stretching vibration of the OH group of lactic acid was observed as a broad band at 3414 cm^−1^. The O-H vibration in glucose appeared at a lower wavenumber (3240 cm^−1^). Band shifting in this area was noticeable after the formation of GluLA, with the O-H vibration appearing at 3358 cm^−1^ in GluLA. This shifting may indicate that the OH- functional groups in lactic acid and glucose take part in the formation of H-bonds in NADESs. It is known that the O-H vibration band shifting indicates an interaction between HBAs and HBDs in NADESs [14,38]. The formation of hydrogen bonds alters the electron density in the bond, which changes the frequency of stretching vibration. Peaks of lactic acid at 1120 and 1211 cm^−1^ can be assigned to C-O-H binding vibration and C-O stretching vibration, respectively. These peaks shifted to longer wavenumbers in GluLA, to 1123 and 1218 cm^−1^. On the other hand, the C=O stretching vibration of LA (at 1718 cm^−1^) did not shift after the formation of GluLA. It should also be noted that during the preparation of GluLA (1:3), 30% water was added in order reduce viscosity and enhance extraction performance. The addition of water may also contribute to the H-binding in NADESs. Vibrational band shifting can be explained by changes in the electron density of the oxygen atoms following interactions with neighboring hydrogen atoms, which may lead to a decrease in the constant force, thus changing the vibrational state.

In the spectrum of GluLA (Figure 9C), peaks related to glucose (Figure 9A) and lactic acid (Figure 9B) were preserved. This indicates that no chemical reaction took place during the formation of NADESs. However, some peaks in NADESs were slightly shifted downfield (designated by arrows in the figure), for example, chemical shifts at 1.227–1.463 of NADESs from 1.353–1.484 in lactic acid, suggesting changes in the chemical environment caused by the formation of hydrogen bonding.

**Figure 10:**
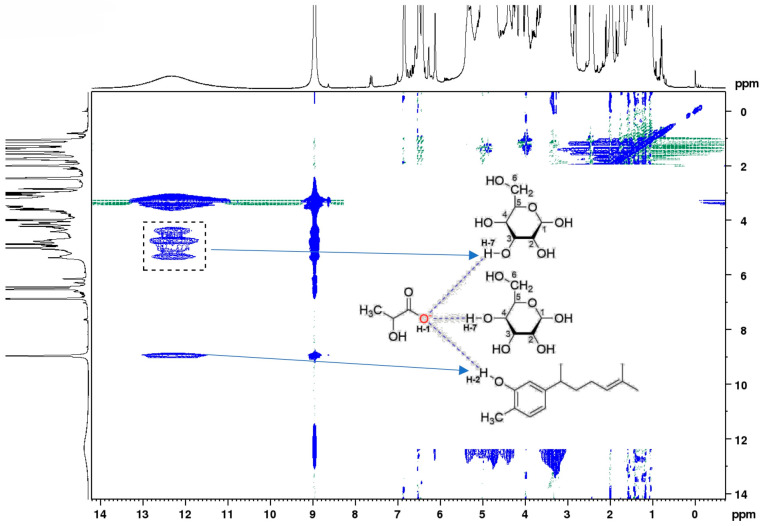
NOESY spectrum of xanthorrhizol in GluLA. Inset: H–H interaction representation.

In the 1H–1H NOESY experiment, the formation of H-bonds can be confirmed by the interaction between the proton of the -OH group at C3 in the glucose and the proton of the -COOH of lactic acid (Figure 10). The target compound xanthorrhizol can be seen interacting via the proton of the -OH functional group with the proton of the -COOH of lactic acid (Figure 10).

## 3. Materials and Methods

### 3.1. Plant Material and Chemical Reagents

The rhizomes of *C. xanthorrhiza* used in this study were obtained from the Research Institute for Herbs and Spices (Balittro, Bogor, Indonesia) and were authenticated by the Biosystematics and Evolution Research Center (BRIN, Cibinong, Indonesia) with specimen voucher number B-609/V/DI.05.07/3/2022. Silica gel 60GF_254_ aluminum plates were purchased from Merck Millipore (Darmstadt, Germany). Lactic acid (85%), malic acid (99%), and glucose (99%) were purchased from Merck (Darmstadt, Germany), while citric acid (99%) was purchased from Sigma-Aldrich (St. Louis, MO, USA). The reference standard xanthorrhizol (>95%) was bought from MarkHerb (Bandung, Indonesia), and curcuminoids (total curcuminoids > 95%) were purchased from Sciyu Biotech Co., Ltd. (Xian, China).

### 3.2. NADES Preparation

NADESs were prepared using a heating and stirring method as described elsewhere [39]. An appropriate amount of the initial components (glucose/organic acid, 1:3), as in Supplementary Table S3, was added into a beaker. The mixture was heated (70–100 °C) and stirred continuously on a hot plate until a clear transparent liquid was obtained. After the formation of NADESs, water was then added (20%, *v*/*v*, of the total NADES volume) to reduce viscosity and aid in the extraction. The liquid was further stirred for 30 min and transferred into a closed glass vessel to be placed at an ambient temperature.

### 3.3. Physical Properties of NADESs

The viscosity of different NADESs was measured using an Anton Paar ViscoQC 300 viscometer (Graz, Austria), using a DG26 spindle type, run for 30 s at 3.00 rpm. The density was determined by weighing 1 mL of each NADES using a Mettler Toledo ML303 analytical balance (Greifensee, Switzerland).

The polarity of the NADESs was determined using a Nile red solvatochromic probe (9-diethylamino-5-benzo[a]phenoxazinone), as described previously [19]. Nile red (20 µL, 0.01 g/mL in ethanol) was added to the NADESs (980 µL). After mixing well, the mixture was scanned in the visible region (400–750 nm) using a Libra S-22 UV-Vis spectrophotometer (Cambridge, UK). The molar transition energy was determined by the following formula:

(3) $$E_{NR}(kcal/mol)=28,591/\lambda_{max}$$

*E_NR_* is the molar transition energy in kcal/mol (results shown in Table 1 were converted into the international unit kJ/mol), whereas *λ_max_* is the wavelength of the maximum absorbance of each NADES. All measurements were conducted at ambient temperature.

### 3.4. Xanthorrhizol Extraction

The extraction of xanthorrhizol from the rhizome powder of *C. xanthorrhiza* was performed with NADESs and ethanol. Ultrasound-assisted extraction (UAE) was used during NADES extraction for screening to select glucose-based NADESs with the highest efficiency. In brief, rhizome powder (0.1 g) was mixed with previously prepared NADESs containing 10–30% water, with a liquid-to-solid ratio of 5–15 mL/0.1 g. Extraction was carried out in a period of 10–30 min. After extraction, the mixture was centrifuged at 2000 rpm for 12 min. The supernatant obtained was filtered and kept at 4 °C until further analysis.

For comparison, ethanol (96%) maceration was conducted following a method reported in the *Farmakope Herbal Indonesia* (2017). Rhizome powder was macerated in ethanol (96%) at a ratio of 1/10 (g/mL) for 18 h with intermittent shaking. After filtration, ethanol was removed under reduced pressure using a Buchi R300 rotary vacuum evaporator (Flawil, Switzerland). The dried extract was kept refrigerated at 4 °C until further analysis.

### 3.5. Extraction Optimization Using Response Surface Methodology

Based on preliminary experiments, three factors that influence xanthorrhizol yield were involved, namely water content in GluLA, solid-to-liquid ratio, and ultrasonic extraction time. The response surface methodology (RSM) with a Box–Behnken Design (BBD) (Design Expert software version 13, Stat-Ease, Inc., Minneapolis, MN, USA) was applied to analyze the interactive effects of these factors. Each factor was determined on three levels, as seen in Supplementary Table S1. A total of 17 experiments were performed, as listed in Table 2. A second-order polynomial equation was applied to generate an experimental model that correlates the responses and three independent variables, i.e., water content, solid-to-liquid ratio, and extraction time, as follows:

(4) $${Y=\beta }_{0}+\sum_{j=1}^{3}\beta_{j}X_{j}+\sum_{j=1}^{3}\beta_{jj}X_{j}^{2}+\sum_{i=1}^{2}+\sum_{j=i+1}^{3}\beta_{ij}X_{i}X_{j}$$

where *Y* is the response variable (xanthorrhizol and curcuminoid contents); *β*_0_, *β_i_*, *β_ii_*, and *β_ij_* represent the regression coefficients of intercept, linear, quadric, and interaction, respectively; *X_i_* and *X_j_* are independent variables; and *k* is a variable number (*k* = 3).

The relationship between the independent variables and responses was examined using analysis of variance (ANOVA) with a significance level of *p* < 0.05, available in Design-Expert version 13.

### 3.6. Determination of Xanthorrhizol Content by TLC Densitometric Analysis

A chromatographic analysis of xanthorrhizol and curcuminoids was conducted based on a TLC method by [40]. Separation was carried out on silica gel 60GF_254_ plates, and was run with a solvent system of dichloromethane/chloroform (4:6). The detection and quantification of marker compounds were conducted on a CAMAG-3 TLC densitometry scanner at wavelengths of 224 and 425 nm for xanthorrhizol and curcuminoids, respectively. A representative TLC-densitometric chromatogram of NADES extract can be seen in Supplementary Figure S1.

### 3.7. Phytochemical Analysis by UPLC-QTOF-MS

A Waters ACQUITY UPLC^®^H-Class System (Milford, CT, USA) was used to generate mass data on the GluLA and ethanol extracts. Chromatographic separation was performed using a Waters ACQUITY UPLC^®^ HSS C18 (1.8 µm 2.1 × 100 mm) (Milford, USA), with the column temperature maintained at 50 °C. Binary solvent systems were used, consisting of 5 mM ammonium formate in water (eluent A) and 0.05% formic acid in acetonitrile (eluent B). A sample volume of 5 µL was injected and eluted at a flow rate of 0.2 mL/min for a total running time of 23 min. The UPLC system was coupled with a Xevo G2-S QTof (Milford, USA) mass spectrophotometer. The collision energy of low- and high-energy functions was set at 4 and 60 eV in positive electrospray ionization (ESI+) mode, over a mass fragmentation range of 50–1200 kDa. The cone and desolvation gas flow rates were 0 and 793 L/h, respectively. Data acquisition and instrument control were conducted using Masslynx software version 4.1.

### 3.8. Determination of Antioxidant Activity

#### 3.8.1. DPPH Radical-Scavenging Activity Assay

To test whether NADESs could be possible radical-scavenging agents, a DPPH assay was applied in accordance with a previously reported method [41]. A volume of 50 µL of sample was added to methanol (50 µL). The DPPH methanolic solution (0.6 mM, 80 µL) was then added into each well. The mixture was left to stand in the dark at ambient temperature for 30 min. The absorbance was read at 515 nm by a Bio-Rad iMark microplate reader (Hercules, CA, USA). Methanol was used as a control. A calibration curve was generated using ascorbic acid as a reference solution (3.13–100 µg/mL). The obtained linear regression equation (y = 0.0085x + 0.9992, *R*^2^ = 0.9729) was used for the calculation of radical-scavenging activity, expressed as the ascorbic acid equivalent (µG AAE/mg sample).

#### 3.8.2. Ferric Reducing/Antioxidant Power Activity Assay

The reducing activity of NADESs was tested using a ferric reducing/antioxidant power (FRAP) method by Jimenez-Alvarez et al. (2008) with some modifications [42]. FRAP reagent was made, consisting of 10 mM 2,4,6-tris (2-pyridyl)-s-triazine (TPTZ) in 40 mM HCl, 20 mM ferric chloride, and 300 mM acetate buffer (pH 3.6) in a 1:1:10 ratio. To a 96-well plate, we added each sample (20 µL), which was then mixed with FRAP reagent (280 µL). The mixture was incubated for 30 min at 37 °C, and the absorbance was read at 595 nm by a Bio-Rad iMark microplate reader (Hercules, CA, USA). A calibration curve was generated using Trolox as a reference solution (12.6–199.8 µg/mL). The resulting regression equation (y = 0.0083x + 0.1454, *R*^2^ = 0.999) was used for the calculation of the reducing activity. The results were expressed as Trolox equivalents (uG TE/gram sample).

### 3.9. DNA Protection Assay

The ability of the optimum NADESs and NADES extract to protect DNA against oxidative damage was evaluated using a DNA protection assay, as reported previously, with some modifications [43]. The pBR322 plasmid DNA (BioLabs, Ipswich, MA, USA) was used as a model, and the •OH free radicals were generated by a Fenton reaction. The reaction mixture (17 µL) contained pBR322 plasmid DNA (5 µL, 5 µG), extracts of different concentrations (5 µL, 1.38–44.18 mg/mL), phosphate-buffered saline (3 µL, 10 mM, pH 7.4), FeSO4 (2 µL, 1 mM), and H_2_O_2_ (2 µL, 1 mM). Following incubation for 30 min at 37 °C, the reaction was terminated by the addition of bromophenol blue buffer dye (Geneaid, New Taipei City, Taiwan), containing bromophenol blue (0.05%), glycerol (50%, *v*/*v*), and EDTA (40 mM). The mixture was then loaded onto 0.85% agarose in Tris/acetate/EDTA gel buffer, to which we previously added GelRed staining dye (Biotium, Fremont, CA, USA). Electrophoreses was run for 60 min at 60 V, and was then visualized under UV light and photographed using a C280 GelDoc Azure biosystem. The results were compared with those obtained for ethanol extract.

### 3.10. Real-Time Bacterial Growth Determination

NADESs were studied for their toxicity profile against *Escherichia coli* ATCC 33218 and *Staphylococcus aureus* ATCC 25923 by measuring real-time bacterial growth according to a reported method by Torregrosa-Crespo et al. (2020) with some modifications [26]. *E. coli* and *S. aureus* were grown in Luria–Bertani (LB) medium overnight at 37 °C in a shaking incubator at 300 rpm. On the following day, the overnight culture (500 µL) was placed in a 50 mL corning tube, together with the LB media (15 mL) and NADES sample (50 µL). The tube was placed in a personal reactor (BioSan RTS 001/001C, Riga, Latvia) and run at 37 °C at 500 rpm. The bacterial growth was monitored by recording the optical density at 850 nm at an interval of 15 min over periods of 15 and 22 h, for *E. coli* and *S. aureus*, respectively.

### 3.11. FTIR Spectroscopy Analysis

The FTIR spectra of glucose-based NADESs were obtained using an Agilent Cary 630 FTIR spectrophotometer (Santa Clara, CA, USA). The measurements were carried out in attenuated total reflectance (ATR) mode, in the range of 4000–650 cm^−1^, at a 4 cm^−1^ spectral resolution, and with the accumulation of 16 scans.

### 3.12. NMR Spectroscopy Analysis

1D and 2D (1H-NMR and 1H-1H NOESY) NMR spectra were obtained for the optimal NADESs and their individual components. The measurement was conducted on a 500 MHz Bruker Avance III 400 (Bruker, Billerica, MA, USA). Chemical shifts were referenced against TMS as an external standard (δ in ppm). NADESs and their starting compounds were prepared in DMSO-d6.

### 3.13. Surface Morphology Characterization

Scanning electron microscopy (SEM) was conducted to study the surface morphology of rhizome residue following NADES extraction. SEM images were obtained using a Hitachi SU-3500 model microscope. The dried rhizome residue was coated with a thin layer of gold. A 20 kV acceleration voltage was used. The images were compared with those obtained for rhizome powder raw material and rhizome residue of ethanol maceration.

### 3.14. Storage Stability Test

To evaluate the effect of storage temperature and time on the stability of xanthorrhizol in the NADES extract, the optimal NADES extracts were prepared and stored at 25 °C in the dark, 4 °C, and −20 °C, respectively, for 90 days. Xanthorrhizol content was analyzed on days 0, 3, 8, 17, and 90 using the HPLC method described previously [44]. The initial xanthorrhizol content obtained on day 0 was considered to be 100%. These results were compared with those obtained for ethanol extract.

## 4. Conclusions

In this study, the NADES GluLA was screened among two other glucose-based NADESs (GluMA and GluCA) for the extraction of xanthorrhizol from the rhizomes of *C. xanthorrhiza*. The prepared NADESs were characterized in terms of their physicochemical properties. All NADESs were viscous and had a higher density than water. The FTIR analysis confirmed the formation of hydrogen bonds between the components forming NADESs, which were the main force that led to the formation of NADESs. The NMR experiments suggested an interaction in NADESs between the -OH group of glucose and the oxygen atom in the -COOH group of lactic acid. The ultrasound-assisted extraction (UAE) of xanthorrhizol was optimized by the response surface methodology with a Box–Behnken Design by optimizing three important factors, namely water content, solid-to-liquid ratio, and extraction time. The predicted yields of xanthorrhizol and curcuminoid contents were close to the experimental results when GluLA-UAE was conducted at optimized conditions. A comparative study with ethanol maceration reveals a higher xanthorrhizol yield and more efficient extraction due to a shorter extraction time with GluLA. The LCMS analysis confirmed the identification of important marker compounds found in GluLA and ethanol extracts, including compounds belonging to diarylheptanoids and terpenoids. The NOESY experiments observed an interaction between xanthorrhizol and lactic acid in GluLA, which probably occurred between the -OH group of the phenolic ring of xanthorrhizol and the oxygen atom of the -COOH group of lactic acid. Moreover, xanthorrhizol extract of GluLA was able to protect DNA from damage, which brings potential health benefits against different degenerative diseases such as cancer and metabolic diseases. Further research should aim at extraction on a larger scale for pilot and industrial tests. Overall, this study recommends the use of a glucose/lactic acid system for the extraction of xanthorrhizol from *C. xanthorrhiza*, which is a greener alternative for application in food and the pharmaceutical fields.

## Figures and Tables

**Table 1:** Physicochemical properties of the studied NADESs.

Sample Name	Polarity (kJ mol^−1^)	Viscosity (mPa s)	Density (kg m^−3^)	FRAP (µG TE g^−1^ NADESs or Solvent) *	DPPH (µG AAE g^−1^ NADESs or Solvent) **
GluLA	198.70	113.9	1.31	29.12 ± 0.09	50.42 ± 0.50
GluMA	199.03	NM §	1.40	9.80 ± 0.13	59.53 ± 0.42
GluCA	207.07	NM §	1.46	31.30 ± 0.97	60.69 ± 2.30
Ethanol	218.07	ND #	0.79	14.22 ± 0.32	6.53 ± 1.93

* µG trolox equivalent g^−1^; ** µG ascorbic acid equivalent g^−1^; § NM (not measurable under the same measurement condition as GluLA); # ND (not determined).

**Table 2:** RSM of independent factors (X_1_, X_2_, and X_3_) for UAE and experimental results for xanthorrhizol and curcuminoid contents (mg/g dried rhizome).

Run	Independent Variables			Responses	
	X_1_	X_2_	X_3_	Xanthorrhizol Content (mg/g)	Curcuminoid Content (mg/g)
1	30	15	20	17.62	6.64
2	10	15	20	4.99	6.02
3	20	10	20	11.85	4.69
4	20	15	10	7.82	6.50
5	30	5	20	1.59	2.28
6	20	15	30	3.53	5.96
7	20	5	10	0	2.39
8	30	10	10	6.03	4.37
9	20	10	20	11.58	4.67
10	10	10	30	2.50	4.81
11	10	5	20	9.50	3.46
12	20	10	20	11.98	4.61
13	30	10	30	5.07	4.46
14	20	10	20	11.99	4.58
15	10	10	10	5.50	4.42
16	20	5	30	0	3.37
17	20	10	20	11.90	4.64

**Table 3:** Phytochemicals identified in GluLA and ethanol extracts of *Curcuma xanthorrhiza* by LC-MS/QTOF. Data were obtained in ESI-positive mode.

Identification	Category	RT (min)	Formula	Measured Mass (Da)	Calculated Mass (Da)	Error (ppm)	MS Fragmentation (*m*/*z*)
Ethanol extract							
Demethoxycurcumin	Diarylheptanoids	10.020	C_20_H_18_O_5_	338.1225	338.1232	−2.1	339, 177, 147
Curcumin	Diarylheptanoids	10.267	C_21_H_20_O_6_	368.1338	368.1362	6.5	369, 285, 177, 149
Zedoarol	Terpenoids	10.723	C_15_H_18_O_3_	268.1151	268.1151	0	269, 247,229
Dihydropyrocurzerenon	Terpenoids	11.074	C_15_H_18_O	214.1436	214.1436	0	215, 159
Isovelleral	Terpenoids	11.777	C_15_H_18_O	232.1528	232.1542	−6.0	233, 215, 197
Curzerenone	Terpenoids	12.087	C_15_H_18_O_2_	230.1382	230.1385	−1.3	231, 213, 149
Curcumene	Terpenoids	12.706	C_15_H_22_	202.1799	202.1800	−0.5	203, 147, 119, 91
Xanthorrhizol	Terpenoids	13.234	C_15_H_22_O	218.1768	218.1749	8.7	219, 201
α-Farnesene/elemene	Terpenoids	13.979	C_15_H_24_	204.1948	204.1956	−3.9	205, 149, 118
GluLA extract							
3-Hydroxy-p-cymene	Terpenoids	1.106	C_10_H_14_O	150.0352	150.0387	−23.2	151,125, 110
ar-Turmerone	Terpenoids	4.923	C_10_H_16_O_5_	217.1080	217.1076	1.8	217, 145, 128
Demethoxycurcumin	Diarylheptanoids	9.936	C_20_H_18_O_5_	338.1241	338.1232	2.7	339, 177, 147
Curcumin	Diarylheptanoids	10.246	C_21_H_20_O_6_	368.1333	368.1339	−1.4	369, 285, 177, 147
Gweicurculactone	Lactone	12.024	C_20_H_26_O_2_	298.1985	2	−8.7	299, 253, 155
Salvinolone	Phenanthrene	12.200	C_20_H_26_O_3_	314.1931	314.1960	−9.2	315, 187
Xanthorrhizol	Terpenoids	13.234	C_15_H_22_O	218.1742	218.1749	−3.2	219, 201, 159, 145
4’-Hydroxy-5,7-dimethoxyflavanone	Flavanone	13.409	C_17_H_16_O_5_	300.1788	300.1804	−5.3	301, 231, 213
Salvigenin	Flavone	13.937	C_18_H_16_O_6_	328.2126	328.2117	2.7	329, 173, 131
Stigmasterol	Steroids	18.415	C_29_H_48_O	412.3792	412.3783	2.2	429, 413, 139

## Data Availability

The datasets generated and analyzed during the current study are available from the corresponding author on reasonable request.

## Supplementary Materials

The following supporting information can be downloaded at: https://www.mdpi.com/article/10.3390/molecules29092093/s1. Figure S1. A Representative of TLC-densitometry chromatogram of *C. xanthorrhiza* extracted by GluLA. Table S1. Independent variables and levels determined on the experimental design. Table S2. Analysis of Variance (ANOVA), factors, and their interaction factors for curcuminoids prediction model. Table S3. The composition of the studied NADES and their abbreviations.
