# Supplementary material for: Ultrasonic-Assisted Extraction of Xanthorrhizol from Curcuma xanthorrhiza Roxb. Rhizomes by Natural Deep Eutectic Solvents: Optimization, Antioxidant Activity, and Toxicity Profiles
Source: Molecules. 2024 May 1;29(9):2093. doi: 10.3390/molecules29092093 (PMC11085723; doi:10.3390/molecules29092093)
Supplement: Supplementary file 1 [file molecules-29-02093-s001.zip › molecules-2944555-supplementary.pdf]

Supplementary Material

Green extraction of xanthorrhizol from rhizomes of *Curcuma xanthorrhiza* Roxb. by natural deep eutectic solvent combined with ultrasonic-assisted extraction

Adelina Simamora[1,2,3], Kris Herawan Timotius[2] , Heri Setiawan[3,4] , Febrina Amelia Saputri[5], Chinthia Rahadi Putri[6], Dewi Aryani[6] , Ratih Asmana Ningrum[3,7] , Abdul Mun'im[3,8*]

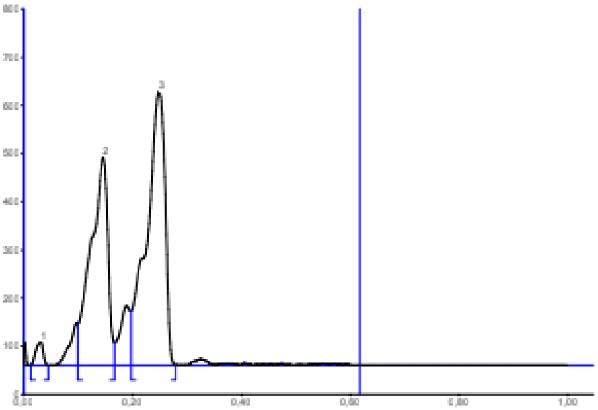

Supplementary Figure S1 A Representative of TLC-densitometry chromatogram of *C. xanthorrhiza* extracted by GluLA.

Supplementary Table S1 Independent variables and levels determined on the experimental design.

Variables	Code	Range and level (xi)		
		-1	0	1
Water content (%)	X_1	10	20	30
Solid-to-liquid ratio (g/mL)	X_2	1/5	1/10	1/15
Extraction time (mins)	X_3	10	20	30

Supplementary Table S2 Analysis of Variance (ANOVA), factors, and their interaction factors for curcuminoids prediction model.

Source	Sum of Squares	df	Mean Square	F-value	*p*-value	
**Model**	24.85	9	2.76	1153.81	< 0.0001	**
X_1- Water content	0.1178	1	0.1178	49.23	0.0002	**
X_2 -Solid-to-liquid ratio	23.16	1	23.16	9678.91	< 0.0001	**
X_3- Extraction time	0.1050	1	0.1050	43.89	0.0003	**
**Interactions**						
X_1X_2	0.8212	1	0.8212	343.12	< 0.0001	**
X_1X_3	0.0233	1	0.0233	9.75	0.0168	
X_2X_3	0.5848	1	0.5848	244.37	< 0.0001	**
X_1^2	0.0061	1	0.0061	2.54	0.1548	
X_2^2	5.084E-06	1	5.084E-06	0.0021	0.9645	**
X_3^2	0.0282	1	0.0282	11.78	0.0110	**
Residual	0.0168	7	0.0024			
Lack of Fit	0.0096	3	0.0032	1.77	0.2914	not significant
Pure Error	0.0072	4	0.0018			
Cor Total	24.87	16				
Std. Dev.	0.0489					
R^2	0.9993					
Adjusted R^2	0.9985					
Predicted R^2	0.9934					
Adeq Precision	114.8548					
Mean	4.59					
C.V %	1.07					

Level of significance: * $p < 0.01$, ** $p < 0.001$

Supplementary Table S3 The composition of the studied NADES and their abbreviations.

Component 1	Component 2	Mole ratio	NADES abbreviation	Appearance
Glucose	lactic acid	1:3	GluLA	Slightly viscous, pale yellowish liquid
Glucose	malic acid	1:3	GluMA	Moderately viscous, transparent
Glucose	citric acid	1:3	GluCA	Highly viscous, transparent

*Following the formation of NADES, each NADES was added with water (20%, v/v).
